# Quercetin as one of the most abundant represented biological valuable plant components with remarkable chemoprotective effects - A review

**DOI:** 10.1016/j.heliyon.2024.e33342

**Published:** 2024-06-20

**Authors:** Alena Vollmannová, Tatiana Bojňanská, Janette Musilová, Judita Lidiková, Monika Cifrová

**Affiliations:** Institute of Food Sciences, Faculty of Biotechnology and Food Sciences, Slovak University of Agriculture in Nitra, Nitra, 94976, Slovak Republic

**Keywords:** Quercetin, Flavonoid, Plant, Nutrition

## Abstract

As a consequence of environmental quality changes as well as changes in our population's lifestyle, there is rapidly increasing variability and many so-called lifestyle disorders, allergies, and food intolerances (also known as non-allergic food hypersensitivity). Unhealthy eating practices, an inappropriate food composition with an excessive energy intake, a high intake of saturated fats, simple sugars, and salt, as well as an inadequate intake of fibre, vitamins, and substances with preventive effects (such as antioxidants), are some of the factors causing this detrimental phenomenon. Enhanced consumption of plant foods rich in valuable secondary metabolites such as phenolic acids and flavonoids with the benefit on human health, food research focused on these components, and production of foods with declared higher content of biologically active and prophylactic substances are some ways how to change and improve this situation. A unique class of hydroxylated phenolic compounds with an aromatic ring structure are called flavonoids. One unique subclass of flavonoids is quercetin. This phytochemical naturally takes place in fruits, vegetables, herbs, and other plants. Quercetin and its several derivates are considered to be promising substances with significant antidiabetic, antibacterial, anti-inflammatory, and antioxidant effects, which could also act preventively against cardiovascular disease, cancer, or Alzheimer's disease.

## 1 Introduction

The diversity and quantity of so-called lifestyle diseases are rising quickly due to detrimental changes in both the modern world's lifestyle and environment [1]. Several factors, including poor eating habits and food composition that includes an excessive intake of energy, a high intake of fats, carbohydrates, and salt, as well as a deficiency in fibre and vitamins, are contributing to this harmful phenomenon. Not only should doctors and nutrition experts strive to improve the situation by educating the public more thoroughly, but food manufacturers should also make a point of offering their products with higher declared concentrations of positively acting and biologically active substances [2]. The subject of global debate in recent years become a polyphenolic substance. Higher plants contain thousands of molecules with a polyphenol structure identified, and edible plants contain several hundred of these molecules. Depending on how many phenol rings they contain and what structural components, like phenolic acids, flavonoids, stilbenes, and lignans, bind these rings together, these compounds can be categorized into various groups [3]. While polyphenols are not essential nutrients, their protective antioxidant properties inhibit oxidative damage of biological systems, which is a reason for their health benefits. The food industry faces a challenge due to the high antioxidant activity of plant phenolic compounds [1]. Massive amounts of various primary and secondary metabolites are produced by plants [4]. Increased production of polyphenols in the plant responds to causes of stress [5]. A class of plant metabolites known as flavonoids are believed to have antioxidant properties and to improve cell signaling pathways. Numerous fruits and vegetables contain these molecules. A special kind of bioflavonoid, quercetin has been the subject of much research in the last thirty years. In 1930, bioflavonoids were first identified [6]. When taken in the right dosage, quercetin has been shown in numerous studies to have a wide range of biological activities, including immunomodulatory, antibacterial, antitumor, neuroprotective, antiallergic, antioxidant, and anti-inflammatory properties. Quercetin can lower blood pressure in hypertensive individuals and lower their chance of developing cardiovascular disease, according to clinical research. Additionally, it might lessen inflammation and muscle damage following exercise [1,7].

## 2 Methodology

Scientific literature review encompasses the results obtained through searching the keywords “quercetin,” “flavonoid,” “characterization,” “nutrition,” “phytochemical,” “plant,” and “biological activity” in major databases such as Web of Science, Scopus, Elsevier, Springer, Google Scholar, up until February 2024.

**Fig. 1:**
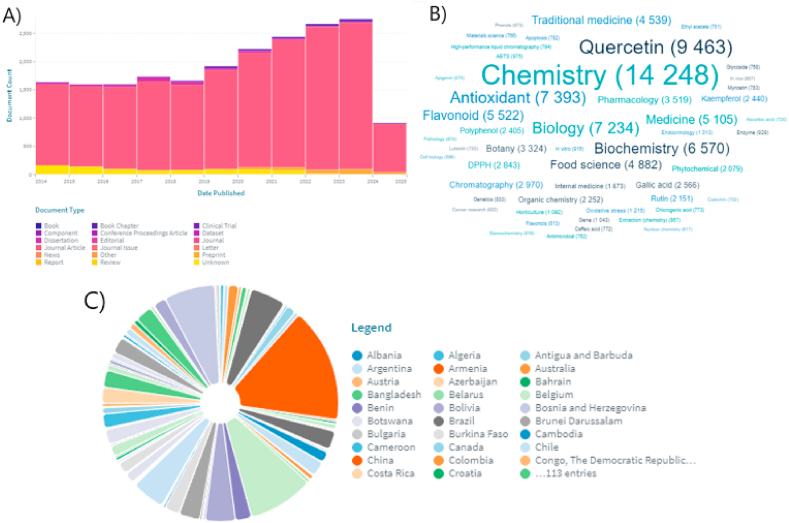
A) Scientific papers (reviewed journal articles only) on quercetin over the past ten years (2014–2024); B) Fields of study; C) Most active countries that have published works on quercetin (Data were taken from Lens.org on May 23, 2024).

All used articles are published and reviewed by several experts in the field. Summarization of the articles took place based on the use of the same methodologies for determining the content of individual components for the objective compilation of this review article (Fig. 1).

## 3 Results and discussion

### 3.1 Flavonoids

**Fig. 2:**
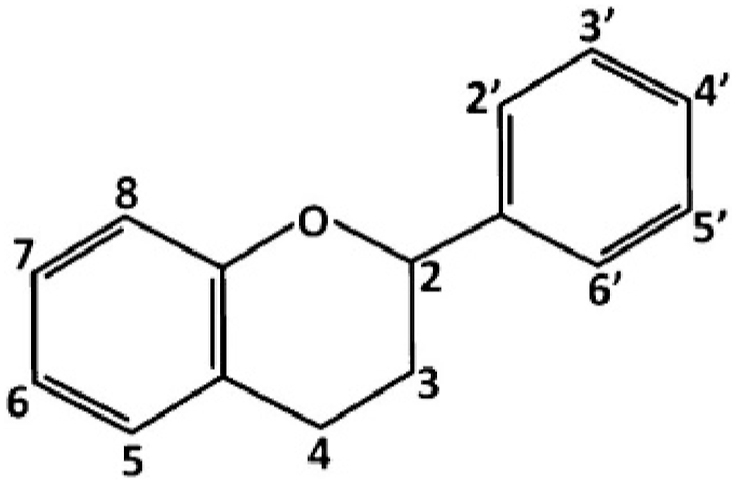
The basic structure of flavonoids [8].

Flavonoids are secondary metabolites present in a range of plants that play a crucial part in the plant's ability to survive by carrying out physiological functions and withstanding negative environmental effects [5]. A vast class of C-15 (C6–C3–C6) secondary metabolites known as flavonoids are found in many higher plants (Fig. 2) as well as some lower plants like algae. A three-carbon unit that may or may not form a third ring (C) connects two aromatic rings (A and B) in the basic skeleton of flavonoids, which have a variety of chemical structures made up of fifteen carbon atoms.

**Fig. 3:**
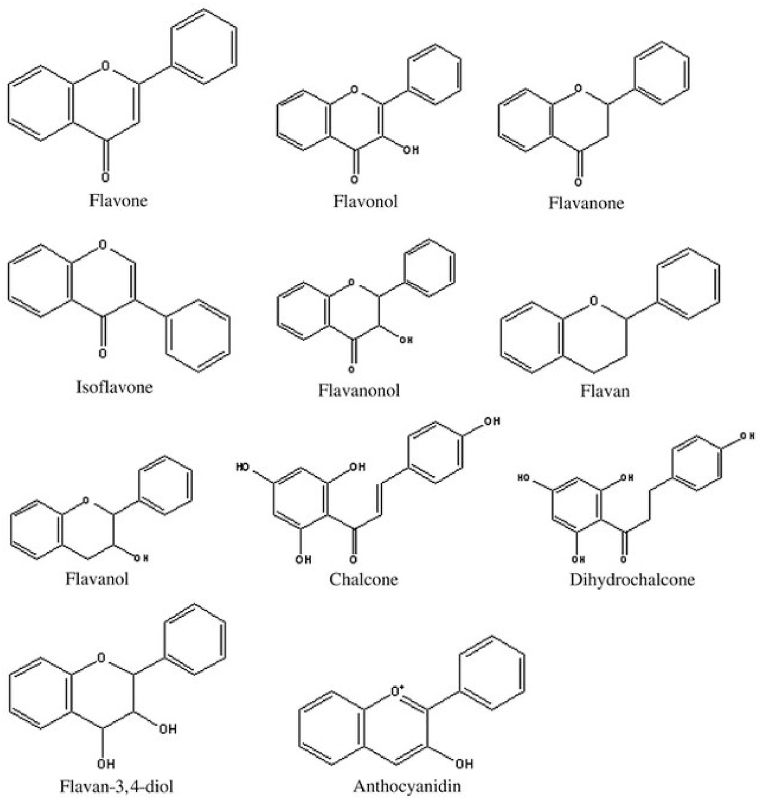
Structure of most common types of flavonoids. Figure reproduced from Ref. [9] with permission from Taylor & Francis.

The majority of these secondary metabolites, referred to as flavonoids (flavone, flavonol, flavanone, flavanonol, flavan, flavanol), can be formed by joining the B-ring to the C-ring at positions C-2, C-3, or C-4. Isoflavones, biflavonoids, flavonolignans, prenylflavonoids, flavonoid glycoside esters, aurones, and chalcones are other classes of flavonoid compounds [[9], [10], [11]]. The general structures of the most common flavonoids are shown in Fig. 3.

The most significant subclass of flavonoids is called flavones. Flavones, also known as glucosides, are abundant in flowers, leaves, and fruits, including red peppers, mint, celery, parsley, and *Ginkgo biloba*. This group of flavonoids includes tangeritin, apigenin, and luteolin. Additionally, diosmetin glycosides are found in edible plants [12]. Whole organs of fruits, vegetables, and grains are rich in flavonols, which are particularly prevalent in green leaves. They are typically found as glycosides based on aglycons, like rhamnetin, myricetin, kaempferol, and quercetin. Among these, the major flavonoids found in edible plants are quercetin 3-*O*-glycosides, which include rutin, isoquercitrin, and quercitrin [13]. Citrus fruits typically contain a type of flavonoid called flavanones, which give the fruit and peel their bitter flavour. Hesperidin in oranges, eriodictyol in lemons, and naringenin in grapefruits are the primary aglycones [14]. Fruits, spices, and tea (*Camellia sinensis*) leaves contain high levels of flavanols, also known as catechins [13].

**Fig. 4:**
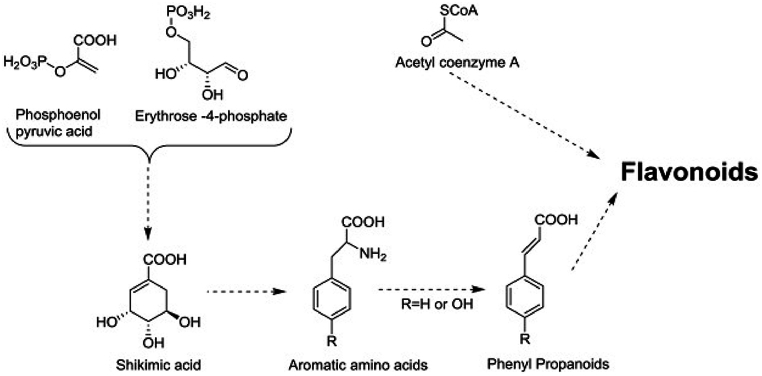
Flavonoids biosynthesis pathway [11].

Plants have two main biosynthetic pathways (Fig. 4) that are used to synthesize compounds based on flavonoids. These comprise the acetate pathway, which acts as a building block for polymeric 2-carbon units, and the shikimic acid pathway, which produces the phenyl propanoids (C6–C3) skeleton [15].

### 3.2 Quercetin

**Fig. 5:**
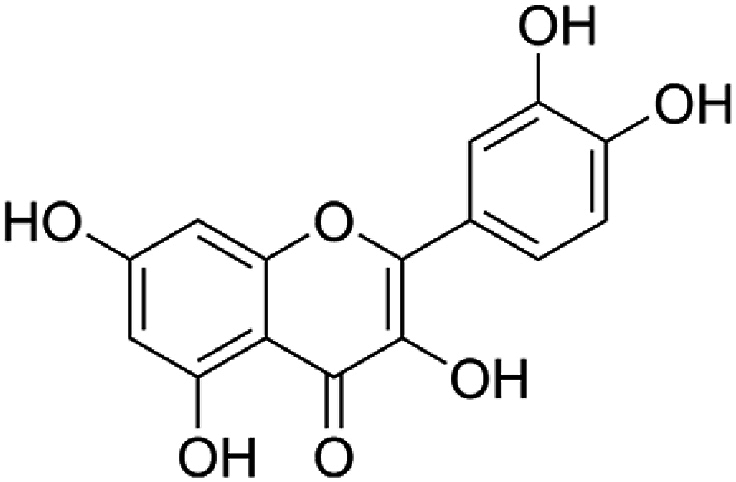
Chemical structure of quercetin.

The compound quercetin, or 2-(3′,4′-dihydroxyphenyl)-3,5,7-trihydrochromen-4-one, is a flavonol, which is present in many fruit and vegetable species. Two benzene rings are joined by a heterocyclic pyrone ring to form quercetin. Its biological activity is attributed to the five hydroxyl groups in its molecule (Fig. 5). These groups also provide the possibility of creating several different derivates [1].

The name quercetin comes from the Latin phrase “quercetum,” which means “oak forest” [5]. Quercetin is the most powerful scavenger of free radicals and has anti-inflammatory, antidiabetic, antiatherosclerotic, antihypertensive, and anti-aging properties [16,17]. With a bitter taste, quercetin is a yellow solid hydrophobic compound that is soluble in glacial acetic acid and only sporadically soluble in alcohol [18,19].

**Fig. 6:**
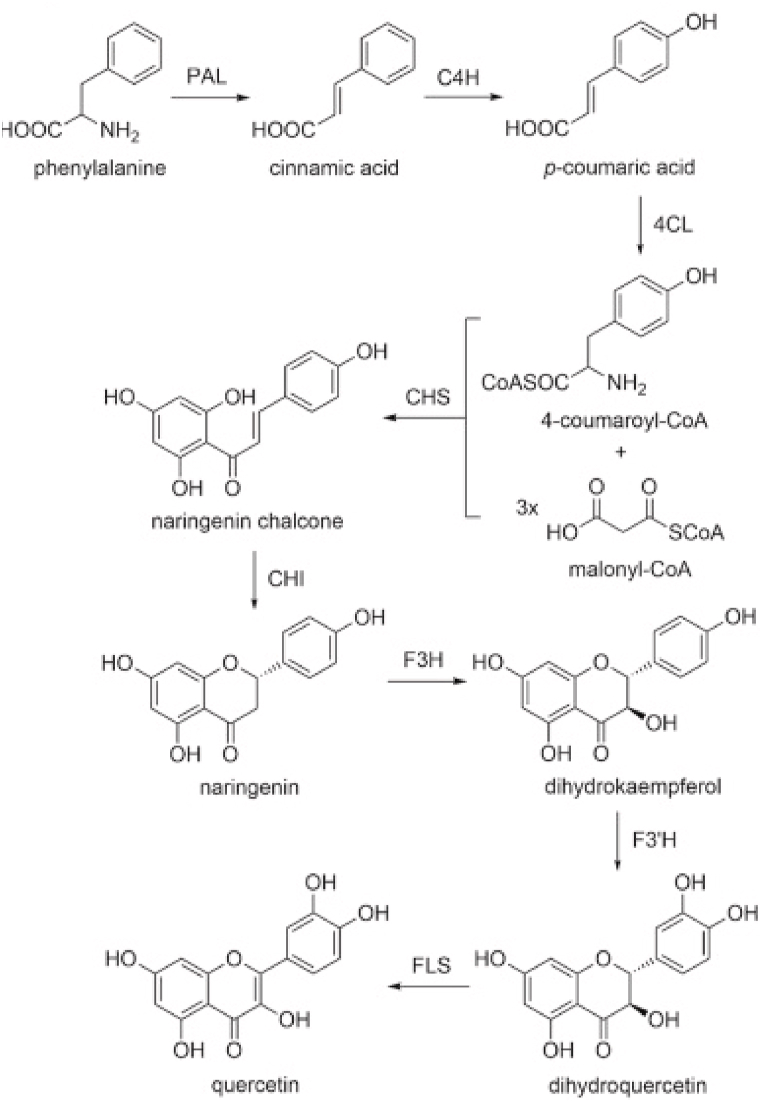
Biosynthetic pathway of quercetin[4].

The metabolic pathway known as phenylpropanoid mediates the biosynthesis of quercetin (Fig. 6). Phenylalanine ammonia-lyase (PAL), cinnamate 4-hydroxylase (C4H), *p*-coumarate: CoA ligase (4-CL), chalcone synthase (CHS), chalcone isomerase (CHI), flavanone 3β-hydroxylase (F3H), flavonol 3′-hydroxylase (F3′H), and flavonol synthase (FLS) are responsible for catalyzing the individual steps.

#### 3.2.1 Quercetin content in plant foods

The beneficial nutritional and physiological effect of polyphenols especially flavonoids is confirmed by a lot of research results. One of the most abundant polyphenolics found in plants is quercetin. Glycoside derivatives are a common form of this aglycone compound found in fruits, vegetables, leaves, and grains [20]. In plants, quercetin rarely occurs as an aglycone. It can be found in different cell types, tissues, and parts of cells. Not only do quercetin and its derivatives shield plants from oxidative stress, but they can also regulate cell growth and differentiation by altering the redox status.

The main flavonoid in our diet daily is quercetin. Vegetables, fruits, and beverages are the main dietary sources of quercetin. Following absorption, the liver and intestines are where quercetin is primarily metabolized [21]. Quercetin is present in diverse plant foods (Table 1). The highest known natural source of quercetin, an atypical KCNQ potassium channel activator, is found in capers [22,23].

**Table 1:** Plant foods high in quercetin.

Plant food	Quercetin content (mg/100 g FW)	Source
capers	1.6–960	[24,25]
Wortleberry	15.8–75.9	[26,27]
Chokeberry	8.9–71	[26,28]
red onion	11.7–68.5	[29,30]
cabbage	38.6–41.4 (red cabbage) 2.61–6.9 (white cabbage)	[31] [32]
cranberry	8.3–25	[26,33]
broccoli	0.03–13.6	[34,35]
buckwheat	0.017–23.1^a^	[[36], [37], [38]]

a content in mg/100 g DW.

Besides many species of vegetables and fruit, also some herbs can be considered important sources of bioactive plant phenolic compounds (Table 2). Herbs and species not only provide an aroma and taste to foods but can help to improve some properties of functional food. They can affect the nutritional quality, appearance, or attractiveness of foods as well as amplify the effect of biologically valuable components and their positive impact on human health.

**Table 2:** Herbs high in quercetin.

Herb	Quercetin content (mg/100 g DW)	Source
dill	40.3–110^a^	[39,40]
fennel	30–178	[[41], [42], [43]]
oregano	10.6–106.1	[44,45]
chili pepper	0.2–11.8	[46,47]
elderberry	0,7–48.5^a^	[48,49]
cilantro	2.3–52.9	[50,51]
watercress	4–29.99^a^	[40,52]
hartwort	29.3–38.9^a^	[38,52]
lovage	2.48–170^a^	[40,53]

a content in mg/100 g FW.

#### 3.2.2 The role of quercetin in plants

Quercetin promotes healthy plant growth and development and helps several physiological processes in plants, including pollen production, photosynthesis, seed germination, and antioxidant machinery. Due to its potent antioxidant properties, quercetin helps plants withstand a variety of biotic and abiotic stresses, including salts, heavy metals, and UV radiation [4]. This flavonoid is found in the chloroplast envelope, most likely in the membrane that surrounds it [54]. Additionally, quercetin is crucial in osmoregulation processes in plants, and as one of the secondary metabolites can be considered an auxin transport regulator [55]. It affects the plant's early-stage adaptation in their local ecosystems [56]. For many years, flavonoids have been extracted using conventional techniques like heat-reflux extraction, hydro distillation, maceration, and soxhlet. Alternative extraction techniques were developed for the extraction of quercetin to address the disadvantages of these methods, which included long processing times, high temperatures, extensive solvent usage, the need for trained personnel to operate, and costs that were prohibitive. Supercritical fluid extraction, microwave extraction, and enzyme-assisted extraction are examples of alternative methods. These techniques are less taxing on extraction time, use fewer organic solvents, and are safe for the environment [57,58].

#### 3.2.3 Important quercetin derivates in plant foods

Plants primarily contain quercetin in highly hydrophilic glycosylated forms, mainly as β glycosides of different saccharides [59]. Quercetin-3,4′-di-*O*-glucoside (q-diglucoside) and isorhamnetin-3-*O*-glucoside (iso-glucoside) belong to quercetin derivates found in edible plants. Quercetin3-*O*-glucuronide (miquelianin), 4′-*O*-methylquercetin (tamarixetin), and 3′-*O*-methyl quercetin (isorhamnetin) are among the most important methylated and glucuronidated quercetin metabolites that are frequently detected in human plasma [60].

Hyperoside (quercetin-3-*O*-galactoside) is the primary ingredient that *Hypericum perforatum* L. contains [61]. Its biological properties include antioxidant activity, reduction in total cholesterol, and elevation in the activity of superoxide dismutase [[62], [63], [64]]. It is reported to have anti-inflammatory, antioxidant, and anti-cancer properties and is present in plants belonging to the genera *Hypericum* and *Crataegus* [65]. Its content in plant-derived products is reported in intervals of 76–1470 mg/kg DW [66].

Tamarixetin (4′-*O*-methylquercetin) has been isolated from *Tamarix ramosissima*. This flavonoid is characterized by xanthine oxidase (XO) inhibitory potential in addition to its superoxide radical scavenging function [67]. Tamarixetin exhibits anti-inflammatory activity, it could strengthen the host immune system and might be used as a particular medication to stop bacterial sepsis [68]. Tamarixetin is also present in the leaves of Blumea balsamifera (L.) DC. (*Asteraceae*), also known as sambong, which has been utilized for thousands of years as medicine in Southeast Asian nations, including the Philippines, China, Malaysia, Thailand, and Vietnam. It contributes to antioxidant activity as well as antityrosinase activities of extract from the leaves of this medicinal plant [69]. Tamarixetin has also in *Azadirachta indica* (neem) a well-known medicinal plant with antiulcer and gastroprotective activity [70] as well as in *Psidium guajava* (guava), the ethanol extract demonstrated 61.3 % inhibition of colon cancer cell line SW480 proliferation [71].

Miquelianin (quercetin 3-*O*-glucuronide) is a primary functional flavonoid found in lotus leaves. It possesses anti-inflammatory, anti-atherogenic, and antioxidant properties, and by dissipating energy, it may be utilized as a new food raw material or nutritional supplement in weight loss products [72]. This flavonoid is derived from *Hypericum perforatum* L., and *in vivo,* pharmacological studies have demonstrated its antidepressant properties [73]. It is present also in *Epilobium* angutifolius L. which has long been applied externally to treat benign prostatic hyperplasia and infections of the skin and mucosa [74,75].

Isorhamnetin (3′-*O*-methyl quercetin) is an important monomethoxyflavonol present in Ginkgo biloba leaves as well as in the fruits of Hippophae rhamnoides L. It is characterized by several pharmacological activities, such as preventing obesity, protecting the heart and brain vessels, preventing tumors, reducing inflammation, and oxidizing damage to organs [76]. It belongs to the main flavonols found in berries [77] and conjugated forms of isorhamnetin have also been found in a variety of foods, including almonds, pears, grapes, apples, and cherries or Opuntia stricta, var. Dillenii fresh fruits [78].

Quercetin-3,4′-di-*O*-glucoside is a flavonol glycoside that can be found in horse chestnut seeds (*Aesculus hippocastanum*) and onions (*Allium cepa*). It is probably a significant factor in onions' anti-proliferative properties [79]. It is believed, that its antiviral activity against influenza virus works by stopping the virus's entry into the cells and inhibiting viral replication. This flavonol glycoside has also anti-inflammatory, antioxidant as well as anticancer activity [80].

Spiraeoside (quercetin-4′-*O*-glucoside) together with quercetin 3,4′-diglucoside are the predominant forms of quercetin is the main flavonoid found in onions (*Allium cepa* cv). These two substances are thought to be protective against cardiovascular disease because they are both powerful antioxidants and free radical scavengers [81]. These two quercetin conjugates are the main flavonols in onion species and make up 80–85 % of the total flavonoid content [82]. Spiraeoside is present also in flowers and fruits of meadowsweet (*Filipendula ulmaria* (L.) Maxim.), which has been extensively employed in the management of numerous illnesses [83].

Quercetin 3-β-D-glucoside is the flavonol glycoside that possesses strong antioxidant, antiviral, and low levels of cytotoxicity. It is believed, that its antiviral activity works well in combating the Ebola virus [84]. It is found in the leaves of Azadirachta indica, which has long been utilized for its therapeutic qualities [85]. Quercetin-3-glucoside is one of the major flavonoid glycosides found in Salicornia herbacea (glasswort), which has numerous pharmaceutical and nutraceutical uses. It is a traditional Asian medicinal plant [86]. In addition to promoting the brain's defense against oxidative stress, quercetin-3-β-D-glucoside improves memory deficits and cognitive impairment brought on by amyloid beta peptides. Its protective effects in neurodegenerative disease could enhance memory and cognitive abilities in people with Alzheimer's disease.

**Fig. 7:**
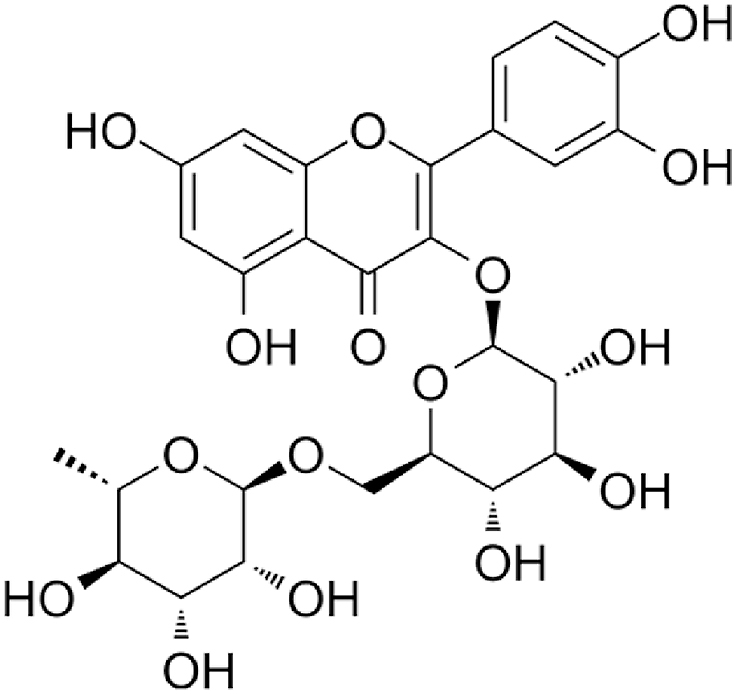
Chemical structure of rutin.

Rutin (quercetin rutinoside) (Fig. 7) is a glycoside consisting of a molecule of flavonol quercetin and a disaccharide rutinose (rhamnose and glucose). The plant *Ruta graveolens*, which contains rutin, is the source of its name. Thanks to its many pharmacological actions (antioxidant, anti-inflammatory, anti-allergic, antiproliferative, and anticarcinogenic), rutin is used in clinical practice to treat capillary fragility and prevent bleeding [87]. It is extensively found in many plants such as vegetables, fruits, and medicinal herbs, especially in passion flowers, buckwheat, asparagus, citrus fruits, and berries. Reputably, buckwheat (*Fagopyrum esculentum*) is among the best foods to consume for rutin [88].

#### 3.2.4 Biological activities of quercetin

##### 3.2.4.1 Antioxidant properties

Quercetin has a very satisfactory antioxidant capacity comparable to curcumin. Its biological effects include inhibition of inflammation and ROS [89]. There is a direct correlation between the quantity of free hydroxyl groups and quercetin's and other flavonoids' capacity for scavenging them. By altering glutathione's role as a reactive hydrogen donor, quercetin produces its antioxidant benefits. Additionally, it was discovered that quercetin enhances antioxidant capacity through signaling pathway modulation. It can scavenge ROS and control how antioxidant-related genes are expressed in A549 cells to reduce oxidative stress [90].

##### 3.2.4.2 Antifungal, antibacterial and antiviral activity

The antimicrobial activity of quercetin includes antifungal, antibacterial, and antiviral effects. The growth of various Gram-positive and Gram-negative bacteria, fungi, and viruses can be inhibited by quercetin. The antimicrobial action mechanism of this substance involves the disruption of cell membrane integrity, inhibition of nucleic acid synthesis, inhibition of biofilm formation, dysfunction of the mitochondria, and inhibition of the expression of virulence factors [91]. Quercetin could also block the receptor-binding domain of S-protein, suggesting not only a receptor blocking but also a virus-neutralizing effect of quercetin on SARS-CoV-2. The theory that quercetin plays a role in host immunomodulation is reinforced by the outcomes of network pharmacology and bioinformatics analysis, making it an even more intriguing option as a COVID-19 inhibitor [92].

##### 3.2.4.3 Anti-inflammatory, immunosuppressive and anti-allergy effects

The active ingredient in a large number of possible antiallergic medications is quercetin plant extract. Its ability to reduce inflammation and allergies has been demonstrated in the treatment of food allergies and respiratory conditions [93]. Quercetin has also remarkable clinical potential and demonstrates strong effects on humoral and cellular immunity [94]. The inhibition of microglial cell activation and the decrease in pro-inflammatory cytokine production account for quercetin's anti-inflammatory properties. Dendritic cells are essential for bridging the gap between innate and adaptive immunity, and [95] found that quercetin effectively inhibited their activation.

##### 3.2.4.4 Prevention against age-related neurodegeneration

The primary risk factor for neurodegenerative disorders, such as Alzheimer's, Parkinson's, and Huntington's diseases, is aging [96]. These disorders affect millions of people worldwide. In addition to age, individual genetics and environmental variables also play a role in the emergence of these conditions. Neuron loss is the main feature of neurodegenerative diseases. Numerous studies both *in vivo* and *in vitro* have documented quercetin's neuroprotective effects. It's still unclear, though, exactly how it works. [97] reported that by modifying and blocking a variety of pathways, quercetin increases the longevity and productivity of neurons.

##### 3.2.4.5 Protective effect against cancer

**Fig. 8:**
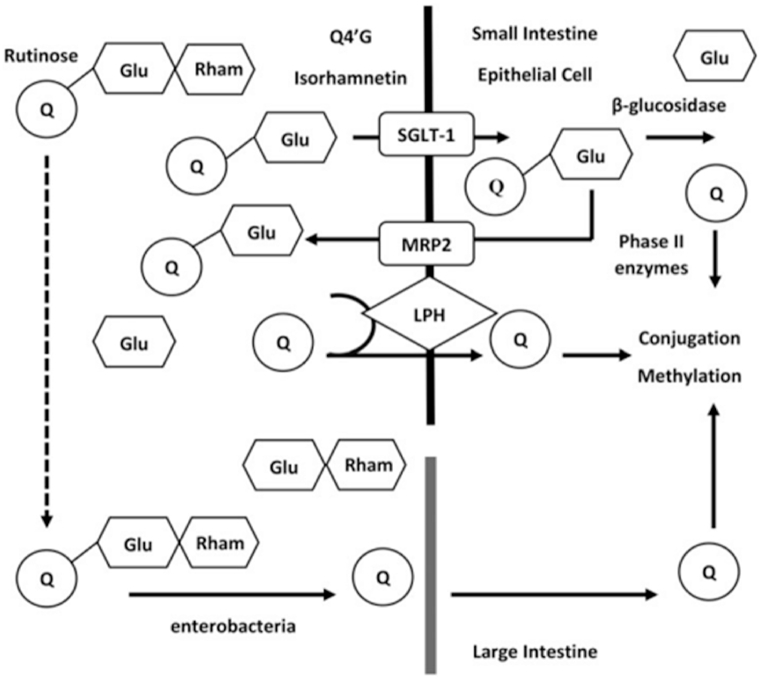
Quercetin absorption from the digestive tract. Figure reproduced from Ref. [101] with permission from Taylor & Francis.

According to Ref. [98], quercetin can reduce cancer cell proliferation and cause various cancer cell lines, such as those from the breast, lung, prostate, and colon, to undergo apoptosis and cell cycle arrest (Fig. 8). Quercetin can block the enzymes that cause carcinogens to activate and can create bonds to cellular receptors and proteins [99]. By altering the molecular pathways of cyclins, pro-apoptotic, and mitogen-activated protein kinase, quercetin induces cell cycle arrest and prevents mitotic processes [100].

##### 3.2.4.6 Solubility of quercetin

Quercetin is a lipophilic bioactive compound that exhibits numerous therapeutic uses. Quercetin, however, is not very useful in the food or pharmaceutical industries because of its poor solubility in aqueous medium. In lipid membranes, quercetin solubilizes more closely to polar groups and exhibits an intermediate polarity between hydrophobic and water-soluble hydrophilic molecules [102]. The number and location of hydroxyl groups in the backbone of the quercetin molecule, as well as the kind and quantity of sugar molecules bonded to it, determine the solubility and transformation of derivatives of quercetin. Other food ingredients that are consumed at the same time as quercetin can also have an impact on its bioavailability [103]. According to the authors [102], the solubility of quercetin is best in nanoemulsions, followed by solubility in oil and only then in water. The glycosyl group in the present nanoemulsions may be the cause of quercetin's increased solubility. Because of their small size, high solubility, high encapsulation efficiency, and long-term stability, nanoemulsions make an excellent drug delivery system. Food-grade nanoemulsions can be used in other food-related applications and offer a good medium for phytochemical solubilization. By preventing quercetin from breaking down too soon in the body, these quercetin nanoparticles have been shown to increase the solubility and stability of quercetin, improving absorption, and cellular absorption, and lowering toxicity [104].

## 4 Conclusion

It has been suggested that dietary polyphenols, such as quercetin, which are secondary plant metabolites, can prevent several chronic illnesses. Quercetin appears to be a valuable substance with possible uses in medication, functional foods, and nutraceuticals. Researchers and food producers face a challenge in creating foods with extra quercetin for functionality or its derivatives. In the production of quercetin-based functional foods, it is necessary to focus on the best positive possible effect on human health. However, while food processing technology advances, care must be taken to ensure that quercetin and its derivatives maintain their structural integrity. A diet richer in quercetin could help prevent several diseases that affect civilization, as well as to the improvement of the overall health status of the current population. It will be crucial to evaluate in the future how these novel quercetin formulations affect the quality attributes of functional foods. Additionally, it is helpful to evaluate the relative effectiveness of various formulations under controlled circumstances to determine which is the most useful and efficient. Ultimately, more *in vivo* research is required to evaluate how these various formulations affect quercetin's bioavailability and bioactivity.

## Ethics declaration

Review and/or approval by an ethics committee as well as informed consent was not required for this study because this literature review only used existing data from published studies and did not involve any direct experimentation/studies on living beings.

## Data availability statement

No data was used for the research described in the article. No data associated with this article has been deposited into a publicly available repository.

## CRediT authorship contribution statement

**Alena Vollmannová:** Writing – original draft, Conceptualization. **Tatiana Bojňanská:** Funding acquisition, Conceptualization. **Janette Musilová:** Validation. **Judita Lidiková:** Validation. **Monika Cifrová:** Writing – review & editing, Visualization.

## Declaration of competing interest

The authors declare that they have no known competing financial interests or personal relationships that could have appeared to influence the work reported in this paper.
